# Simulation analysis of high-throughput oyster cryopreservation at three scales of production

**DOI:** 10.1007/s10499-023-01139-y

**Published:** 2023-06-05

**Authors:** Sarah Bodenstein, Isabelina Nahmens, Brian R. Callam, Terrence R. Tiersch

**Affiliations:** 1 Aquatic Germplasm and Genetic Resources Center, School of Renewable Natural Resources, Louisiana State University Agricultural Center, Baton Rouge, LA 70820, USA; 2 Department of Mechanical and Industrial Engineering, Baton Rouge, LA 70803, USA; 3 Aquaculture Division, Maryland Department of Natural Resources, Annapolis, MD 21401, USA

**Keywords:** Cryopreservation, Germplasm repository, Simulation modeling, Oyster aquaculture, Scaling production

## Abstract

Cryopreservation and germplasm repositories offer a variety of potential benefits to aquaculture industries. Despite this, no comprehensive repository systems exist for any prominent aquaculture species. A species that could greatly benefit from the use of cryopreserved sperm and repository storage is the eastern oyster, *Crassostrea virginica*. High-throughput cryopreservation protocols already exist for this species, and the easy transport of frozen sperm could facilitate selective breeding programs that address pressing challenges currently faced in the industry, such as mortality due to low-salinity conditions. This study addressed the gap between cryopreservation protocols and repository development in the oyster industry by creating simulation models to evaluate cryopreservation needs at three different scales of production. The effects of high-throughput device options and three key parameters (straws per oyster, batch size, and number of operators) on production capacity, time, and cost were evaluated. Recommendations for decisions concerning cryopreservation pathways and repository creation were given at each scale of production. Relative values of broodstock, juvenile oysters, and oyster sperm sold at hatcheries were also discussed. In general, repositories operating at higher production levels benefited from the economy of scale, could use automated high-throughput equipment options, and could hire more labor without drastically increasing production costs.

## Introduction

Oyster aquaculture dates back centuries to the time of the Roman empire and remains an important industry today (Botta et al. 2020). Throughout that history, technological advancement has substantially shifted the landscape of oyster aquaculture. The introduction of polyploid animals allowed farmers to harvest earlier in the season or harvest during spawning months (Allen and Downing 1986). The popularization of off-bottom gear along the US Atlantic coast allowed the industry to shift focus to the high-end, half-shell market (Petrolia et al. 2022). Cryopreservation and repository storage are proven technologies for other commodities but are new to oyster aquaculture and this study will attempt to anticipate the changes that these technologies will have in the future.

Repositories are rigorously maintained collections of cryopreserved (frozen) germplasm samples and associated information, such as phenotypic and genetic data (Hu et al. 2015). Through cryopreservation and repository storage, farms, hatcheries, and stock centers can safeguard valuable genetics from losses due to disease, accidents, and natural disasters while simultaneously reducing the number of live populations that must be maintained (Yang et al. 2021). In addition, repositories enable selective breeding programs and rapid genetic improvement by providing ease of transport of germplasm (e.g., cryopreserved sperm) across hatcheries (Curry 2000; Moore and Hasler 2017; Yang et al. 2012). The dairy industry first began using cryopreserved semen from repository collections for artificial insemination in the 1950s, and today, the practice is established worldwide (Moore and Hasler 2017).

In the USA alone, more than 17 million breeding units (typically packaged within 0.5-ml French straws) of dairy bull semen were sold in 2021 (National Association of Animal Breeders 2022). The use of cryopreserved semen has enabled precise breeding practices that have facilitated significant increases in milk production, health, and fertility (Carvalho et al. 2022; Moore and Hasler 2017). The development and integration of germplasm repositories for eastern oysters (*Crassostrea virginica*) into aquaculture operations could increase production in a similar way. Today, farmers face oyster mortality due to challenges such as disease and unsuitable water quality conditions including low salinity (Casas et al. 2018; Matt et al. 2020). Breeding of oyster lines that are tolerant to these challenges and distributing their cryopreserved sperm across hatcheries could decrease mortality and allow breeders to work collaboratively to advance genetic improvement, similar to the advances seen in the dairy industry (Moore and Hasler 2017). Despite these potential benefits and more than 30 years of cryopreservation research, repositories are not widely used in oyster aquaculture or any aquaculture industry (Paniagua-Chavez and Tiersch 2001; Yang et al. 2012, 2021).

Aquaculture of the eastern oyster is an important part of the US coastal economy with an industry valued at over $130 million in 2018 (farm gate value, Perdue 2019). Oyster hatcheries produce larvae which are raised until becoming juveniles that are large enough to be sold to farmers and grown in field sites (Wallace et al. 2008). Oysters have traditionally been grown sub-tidally, or “on-bottom,” but “off-bottom” aquaculture (where oysters are suspended in the water column using various types of equipment) has become increasingly popular, particularly along the Atlantic coast (Walton and Swann 2021). To offset the higher investment of using “off-bottom” equipment, many farmers have turned to growing triploid (3N) oysters (Petrolia et al. 2022). Triploids have three sets of chromosomes instead of the usual two (diploids, 2N) and as a result have reduced gametogenesis, faster growth, and better meat quality than diploids. Due to their inability to reproduce, hatcheries produce triploids by crossing tetraploid (4N) males with diploid females (Dong et al. 2005). The popularity of triploids and the resources required to produce tetraploid lines make tetraploid germplasm valuable to hatcheries. Hatcheries also have valuable germplasm in the form of selectively bred diploid lines, created to be disease resistant (e.g., lines resistant to *Perkinsus marinus*, S. Casas et al. 2017 and *Haplosporidium nelson*, Haskin and Ford 1979) or resistant to low salinity (McCarty et al. 2020). Low-salinity resistance is a trait of importance because although eastern oysters can tolerant a wide range of salinities, long periods of low salinity (< 5 ppt) can result in significant mortality (La Peyre et al. 2013; Rybovich et al. 2016). Thus, it is problematic that despite the value of these oysters, their genetic resources are not routinely stored in germplasm repositories.

Integration of repositories into an aquaculture system is a complex task that requires analysis of numerous interacting resources and communities. Understanding those interactions is crucial for repository development in aquaculture, and simulation models are a useful tool for doing so. A specific type of model, discrete-event simulation models, is capable of emulating large-scale production systems by modeling all the events (steps) and resources that occur or are used in a system throughout time (Allen et al. 2015; Gittins et al. 2020; Schriber et al. 2013). While this type of modeling has historically been used in the manufacturing sector (Robinson 2005), it has also been applied to other industries such as healthcare (Oueida 2016).

Discrete-event simulation models have utility to benefit aquaculture as well, particularly concerning the slow adoption of cryopreservation technology and germplasm repositories. The goal of this study was to respond to the challenges of repository development in oyster aquaculture by evaluating cryopreservation needs at different scales of production. The objectives were to (1) characterize two existing models of high-throughput oyster cryopreservation, (2) evaluate the effect of three parameters (straws per oyster, batch size, and number of operators) on the throughput, time in system, and operating cost of the models, (3) develop scaling and breakeven point analyses to analyze the models at the different scales, (4) make recommendations for high-throughput cryopreservation pathways at each scale of production, and (5) describe the relative values of broodstock, juvenile oysters sold by hatcheries (oyster “seed”), and oyster sperm to provide context for uses of germplasm resources.

## Methods

### Introducing the cryopreservation models

The two cryopreservation models, Models A and E, used in this study were initially developed in a previous study (Table 1, Bodenstein et al. 2022). Time study data of the steps comprising the cryopreservation protocol (Yang et al. 2012) were used to generate discrete-event simulation models in the software Simio (2021,2022 v14.230, Simio LLC, Sewickley, PA). To simulate real-world conditions, four key parameters were set to baseline values: the workday (run time) was 8 h, the number of operators was one, the straws per oyster was 45, and the batch size was five. These values were chosen to reflect the working conditions and constraints of a single operator cryopreserving oyster sperm (Bodenstein et al. ). The number of straws per oysters was chosen to preserve a sufficient quantity of samples for future research (divisible by 15 so that a sorting funnel could be used) without overwhelming the operator. The terms “straws” refers to the freezing container used: French straws. French straws are cylindrical, “straw-shaped” containers with a cotton and polyvinyl alcohol powder seal on one end (0.5-mL volume, 133 mm length, 2.5 mm interior diameter; https://www.imv-technologies.com/product/classic-straws). Batch size refers to the number of oysters and associated French straws that could be frozen simultaneously due to time-sensitive constraints of the cryopreservation process. When diluted sperm samples were mixed with cryoprotectant, a 20-min equilibration window began that was set to maximize chemical and physical phenomena (e.g., diffusion and dehydration) and minimize toxicity (Tiersch and Green 2011; Yang et al. 2012). During that 20-min window, samples had to be packaged into French straws, placed on a freezing rack, and inserted into the freezer unit before time expired. This limited the number of samples (i.e., batch size) that could be frozen at one time. The number of straws per oyster remained constant because freezing a sufficient quantity of samples was a priority. Associated costs (i.e., equipment, supplies, and labor) were also inserted into Models A and E.

Five models, Models A–E, were created and evaluated in Bodenstein et al. (2022), each with different equipment or devices implemented that altered model production efficiency (throughput) and cost. Models A and E were selected for analysis in the current study because they represented the cryopreservation pathway under baseline conditions (Model A) and the pathway with all device improvements incorporated and enhanced throughput (Model E). Models A and E contained the same steps, logic rules, and baseline conditions. They also used the same devices (tools and equipment) to process samples except for the specific options used in the steps where sperm motility was measured and the step where straws were sorted into storage containers after freezing. Computer-assisted sperm analysis equipment (CASA, CEROS II^™^ Animal—Hamilton Thorne Ltd, Beverly, MA) was used to quantify sperm motility in Model E instead of the microscope and Makler^®^ counting chamber used in Model A. Additionally, a custom designed 3-D printed funnel was used to sort 15 French straws at a time into daisy goblets after freezing was completed in Model E instead of individually sorting straws with forceps (Model A). These changes were reflected in new time distributions for the steps in Model E where motility assessment and sorting of straws took place. These alternate devices were substituted into Model E to improve throughput because previous work identified these steps as major process constraints (Bodenstein et al. 2022). In the current study, Model A will be referred to as the “baseline model” and Model E will be referred to as the “enhanced model”.

The major outputs analyzed for both models were throughput, time in system (TIS), operating cost, cost per oyster, and total cost. *Throughput* was the number of oysters fully processed during the 8-h workday. *Time in system* was the average amount of time it took to process an individual oyster (i.e., the average each oyster spent in the model). *Operating cost* was the average cost of supplies and operator wages to process all oysters in a single batch, excluding *capital costs* (the one-time cost of equipment). *Cost per oyster* was the average *operating cost* of processing of each oyster. Finally, *total cost* was the cost to process all oysters in a single batch including *operating* and *capital costs*. The baseline value for the workday (run time) was 8 h, the number of operators was one, the straws per oyster was 45, and the batch size was five.

### Evaluating parameter effects

Linear regressions were used to determine relationships among three parameters (straws per oyster, batch size, and number of operators) and three output statistics (*throughput*, *operating cost*, and *time in system)* for the baseline and enhanced models. For both models, the *throughput*, *operating cost*, and *time in system* were calculated based on a range of values for straws per oyster (5–300), batch size (1–7), and number of operators (1–6). Ten replicates were run for each models under every condition. The workday remained consistent at 8 h for all comparisons.

### Scaling and break-even point analyses

To assess how the baseline and enhanced models would perform at different scales of production, a series of scenarios were created. Scenario 1 required that 50 oysters be processed in 1 year. Scenario 2 required that 1000 oysters be processed in 1 year, and scenario 3 required that 10,000 oysters be processed in 1 year. In addition, the number of operators in each scenario was tested at 1, 2, 3, 5, or 10 to analyze the effect varying this parameter had on results, such as percent processing failure, required processing time, breakeven price, and breakeven times (all results defined in the next paragraph). The number of straws was held constant for all scaling analysis scenarios, and each scenario required that 45 straws would be frozen per oyster. The baseline and enhanced models were run using each scenario. These scenarios were not run within an 8-h workday, but rather continued to run until all required oysters from a given scenario had been processed.

The *percent processing failure* statistic represented the number of oysters per year that were not successfully cryopreserved. Oysters “failed” and were discarded from the system if freezing had not begun before the 20-min equilibration time ended. At the end of the simulation, the ratio of failed oysters and oysters that were fully processed was calculated. To calculate *percent processing failure*, a timer was placed at the beginning of the step where equilibration time began in both models. This timer was triggered each time a batch of oysters entered the step. A second timer was in the step where freezing began (after the 20-min equilibration time had ended). If longer than 20 min passed between a batch triggering the first and the second timers, the batch was discarded and all oysters within that batch were considered “failed.” The required processing time was the number of 8-h workdays needed to process all oysters under a given scenario.

The cost per straw, breakeven price, and breakeven times were also calculated to perform a breakeven point analysis. Such analyses are commonly used in business to determine the amount of revenue needed to cover *total costs*, which includes *operating and capital costs* (Childress et al. 2018; Gandonou et al. 2006). The *cost per straw* was the cost needed to cover the *operating costs* to produce a certain number of straws and was calculated by dividing the *operating costs* by the total number of straws produced. The *breakeven price* of straws, the *cost per straw* needed to cover *total costs*, was calculated using the equation BeP = (CC + (OC × t))/(NS × t), where BeP is the *breakeven price* of straws, CC is the *capital cost*, OC is the *operating cost*, t is the time in years (a 1-year time period was allotted), and NS is the total number of straws produced. A 1-year time period was selected when calculating *breakeven price* to demonstrate a “worst case” scenario where profits are required by the second year.

*Breakeven price* could also be viewed as the cost to produce one straw, including initial *capital costs*. The *breakeven time* was the time, in years, required to pay off initial *capital costs* and *operating costs* for each year of production, based on the price of a straw. The *breakeven time* was calculated using the equation BeT = (CC/(SP − CP))/NS, where BeT is the *breakeven time*, CC is the *capital cost*, SP is the straw price, CP is the average cost to produce a straw, and NS is the total number of straws produced. There currently are no industry pricing structures for frozen oyster sperm; therefore, three fixed straw prices were selected based on average prices of semen from dairy bulls. The average minimum price reported was $14, the average price for “non-sexed” (does not guarantee female offspring) semen was $27, and the average price of all dairy bull semen was $57 (Carvalho et al. 2022). Based on these values, the three fixed straw prices selected for this study were $15, $30, and $60.

In addition to fixed straw prices, the potential gross straw value was calculated. The *gross straw value* was calculated using the equation: *Gross Straw Value* = (number of oyster seed per straw × oyster seed price point). The number of 6–10 mm oyster seed that could be produced based a single straw of frozen sperm was calculated using actual oyster fertilization and survival rates, and the number of straws required to fertilize a specific number of eggs based on discussions with hatchery operators (B. Callam pers comm; F.S. Rikard pers comm; Wallace et al. 2008; W.C. Walton pers comm; Table 4 in the Appendix). The average price to purchase a single diploid, 6–10 mm oyster seed from a hatchery was calculated to obtain the straw price point (Oyster Seed Holdings Inc. 2021; University of Maryland 2022). Finally, the *net straw value* was calculated by subtracting the *breakeven costs* (production costs) from the *gross straw value*.

### Relative Values of Broodstock, Juvenile Oysters, and Sperm

A table was generated to describe the relative values of broodstock oysters, juvenile oysters sold by hatcheries (oyster “seed”), and oyster sperm. Oyster seed order forms from commercial hatcheries were analyzed to compare the prices of different types of seed offered, such as seed from diploid, triploid, “wild” (the line had undergone no breeding program), and selectively bred lines. The relative values of seed were assessed from on the perspective of commercial oyster producers, where triploid seed would be more valuable (compared to diploids) due to faster growth rates and selectively bred lines would be more valuable (compared to wild lines) due to valuable characteristics, such as disease resistance. Next, the relative values of broodstock oysters and frozen sperm were assessed based on the perspective of hatchery managers who would sell the resulting seed to producers. A hatchery manager with over 5 years of experience was consulted (Callam, pers. comm.) and the previously defined seed value also influenced broodstock and sperm value.

## Results

### Parameter effects

**Fig. 1:**
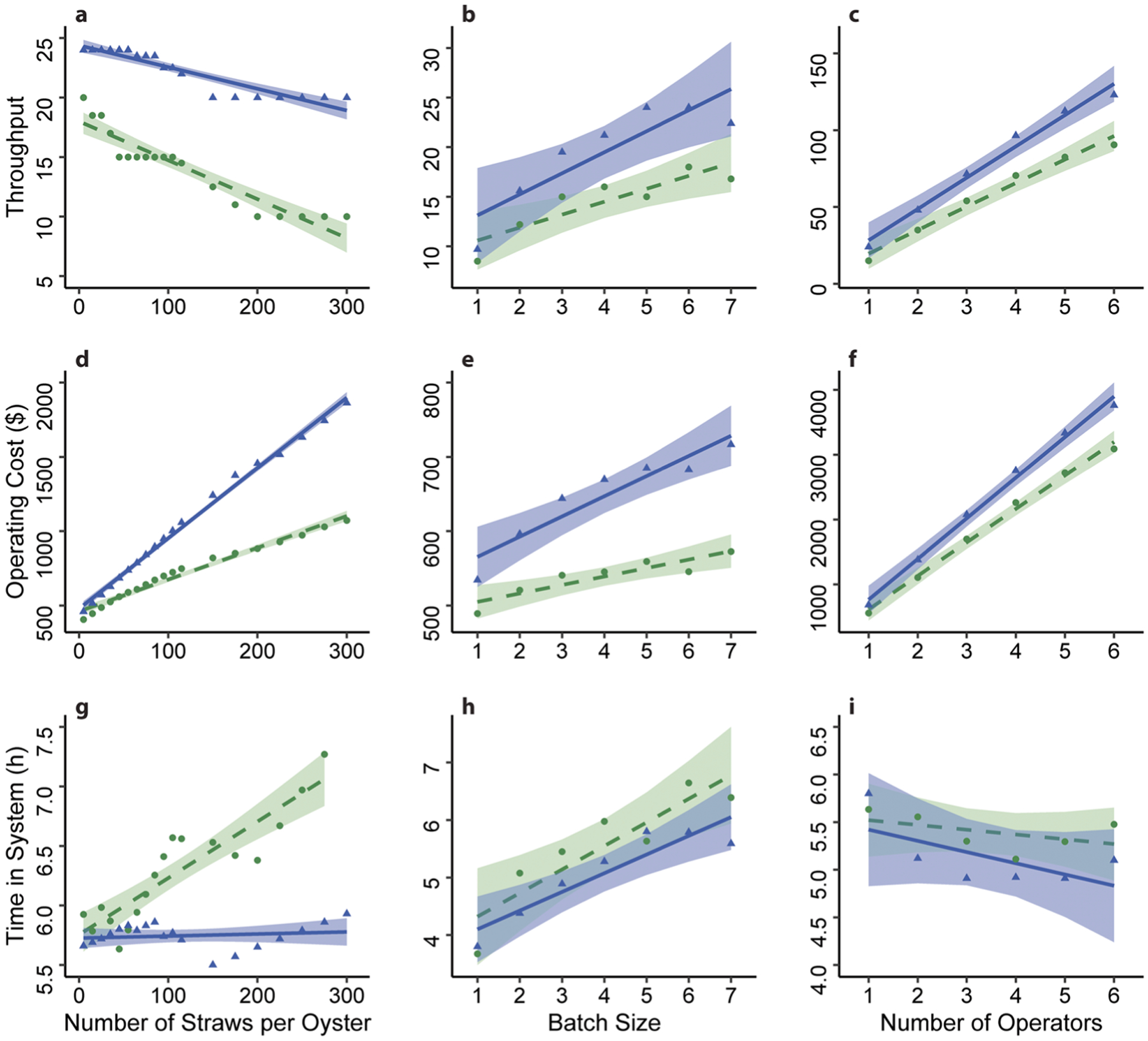
Linear regressions analyzing the effects number of straws per oyster, batch size (number of oysters), and number of operators (arrayed along the *X*-axis) on the throughput (number of oysters per 8-h day), operating cost, and time in system (arrayed along the *Y*-axis) in models A (dashed lines) and E (solid lines). Circles represent data points from the baseline model and triangles represent data points from the enhanced model. Shaded areas indicated 95% confidence limits for the baseline model and enhanced model regressions

The straws per oyster, the batch size, and the number of operators affected the *throughput*, *time in system* (*TIS*), and *operating cost* in the baseline and enhanced models (Fig. 1). As the batch size and the number of operators increased so did the *throughput* and *operating cost* in both models (Fig. 1b, c, e, and f). As the straws per oyster increased, *throughput* decreased while *operating cost* increased in both models (Fig. 1a and d). While the relationship trends between the three parameters (straws per oyster, the batch size, and the number of operators) and the *throughput* and *operating cost* were the same in the baseline and enhanced models, the magnitude of their effects (the slopes) was significantly higher in the enhanced model (generalized least squares regression, *P* ≤ 0.2 for all comparisons, Fig. 1c–f). The exception being the effect of straws per oyster on *throughput*, where the effect was stronger in the baseline model than the enhanced model (Fig. 1a). When examining how *TIS* was affected by the three parameters, the baseline model produced a larger magnitude of effect than the enhanced model (Fig. 1g–i), although the effect was only significant for number of straws and *TIS* (generalized least squares regression, *P* < 0.001, Fig. 1g). In fact, as straws per oyster increased, the *TIS* of the enhanced model was not affected while the *TIS* of the baseline model increased an average of 0.6 min for each straw added (*P* < 0.001, Table 2).

### Scaling and break-even point analyses

#### Baseline conditions

Under baseline conditions (45 straws per oyster, batch size of five, one operator), the percent processing failure values were similar (< 10% difference) for the baseline models across all scales of production (mean of 25% ± 0.7 SD). The percent processing failure values were also similar (< 10% difference) for the enhanced models across all scales of production (mean of 11% ± 0.4 SD, Table 1). The required processing times (number of 8-h workdays) increased as the scale of production increased (when the number of operators was held constant) for both models. The enhanced model, however, had shorter processing times than the baseline model compared within the same production scales with the same number of operators.

*Breakeven prices* (price required for a unit of germplasm to pay off *capital* and *operating costs* in 1 year) decreased at larger scales of production (Table 1). *Breakeven times* (years to pay off *capital and operating costs* based on fixed straw price) followed the same pattern, decreasing at larger scales of production (Table 1). In addition, as the straw price increased, the *breakeven time* decreased for both models at all production scales. The baseline and enhanced models had similar (< 5% difference) *breakeven times* at each scale of production for all straw prices.

#### Adjusting the number of operators

**Fig. 2:**
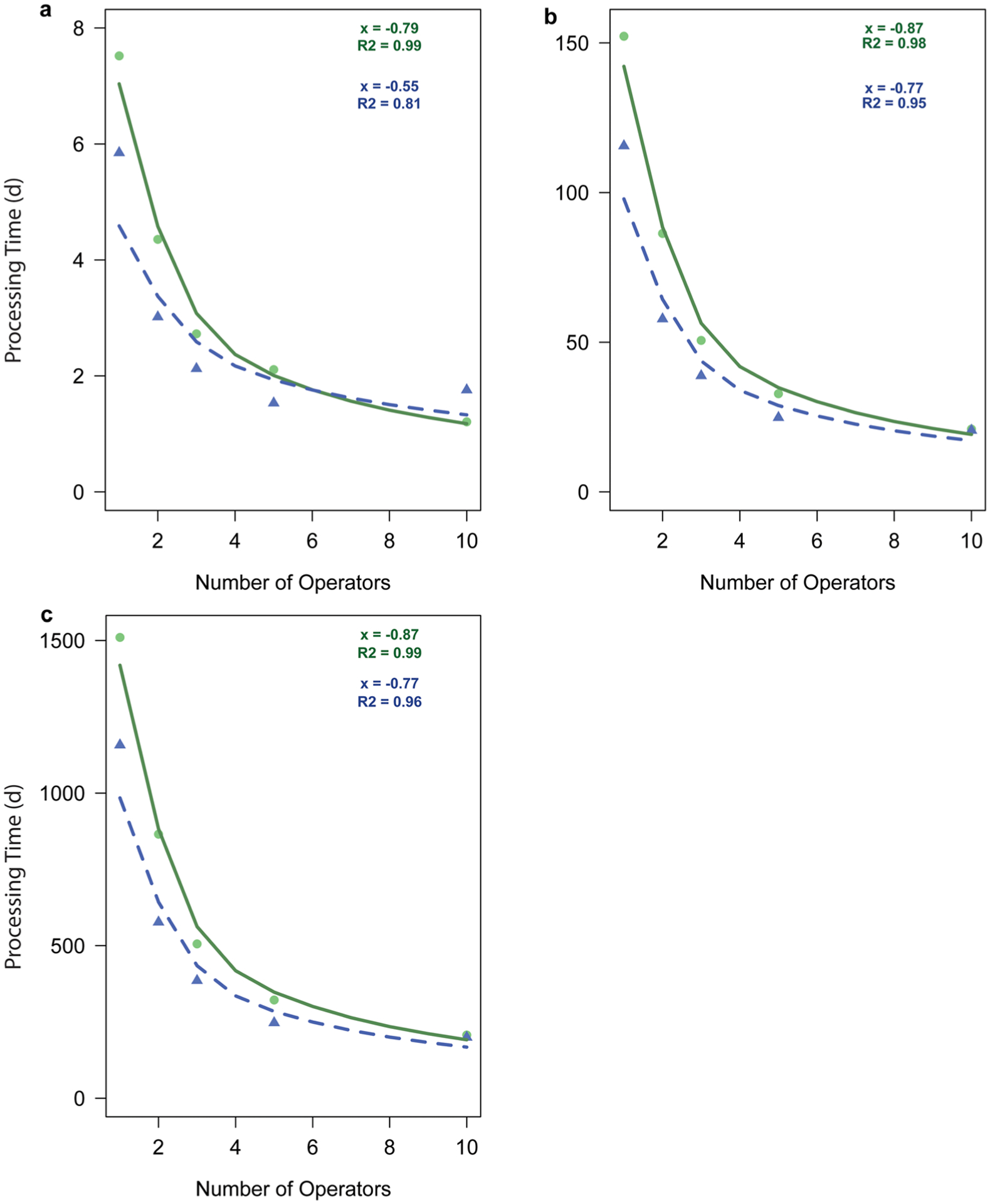
Plotted power functions of the relationship between the number of operators and the required processing times at three scales of production (A: 50 oysters per year, B: 1000 oysters per year, C: 10,000 oysters per year) for the baseline model (solid line) and the enhanced model (dashed line). Circles represent data points from the baseline model and triangles represent data points from the enhanced model

When more operators were assigned to the baseline and enhanced models, the *percent processing failure* values and required processing times generally decreased (within the same production scale), while the *breakeven prices* generally increased (Table 1). When more than one operator was assigned to a model, the *percent processing failure* values dropped to zero at all scales of production. Within the same the same scale of production, as the number of operators increased, the processing time decreased. A power regression was fit to explain this relationship using the equation *y* = *ax*^*b*^, where *y* was the response variable (processing time), *x* was the predictor variable (number of operators), *a* was the intercept, and *b* was the slope (Fig. 2). Initially, increasing the number of operators decreased the processing time at a faster rate. For example, processing time decreased by one day when the number of operators increased from one to two in the baseline model at the smallest scale of production. However, as more operators were assigned, the rate at which processing times decreased was slower. For example, processing time decreased by 0.03 day when the number of operators increased from nine to ten in the baseline model at the smallest scale of production. The slopes of the power functions (the b values) were significantly steeper in models operating at the medium and large scales of production (1000 and 10,000 oysters per year) than in models operating at the smallest scale (50 oysters per year, generalized least squares regression, *P* ≤ 0.008 for all comparisons, Fig. 2). The slopes of the power function in the models operating at the medium and large scales were not significantly different (generalized least squares regression, *P* = 0.9). When comparing the baseline and enhanced models within the same scale of production, the baseline model always had steeper slopes. Therefore, increasing the number of operators had a stronger effect on processing times at higher scales of production across both models and a stronger effect on the baseline model across all scales of production.

For the baseline and enhanced models at medium and large scales of production, the number of operators had an effect on the *cost per straw* and the *breakeven price* of a straw, although the effect was small (general linear model, *P* = 0.04 or less for all cases). As the number of operators increased, so did the *cost per straw* and *breakeven price*; however, the price increase was < $0.1 as the number of operators increased by one (Table 1). For the baseline model at the small scale of production, as the number of operators increased from 1 to 10, the *cost per straw* increased by $0.02 (general linear model, *P* < 0.001); however, the number of operators did not produce a significant increase in the *cost per straw* for the enhanced model, at the small scale (general linear model, *P* = 0.05). Across all scales of production, the *costs per straw* values were similar (< 5% difference), except for the baseline model at the small production scale where there was an increase of 37% as the number of operators increased from 1 to 10 (Table 1). When looking at *breakeven price* at the small scale of production, no significant relationship was found between the number of operators and the *breakeven price* for the baseline and enhanced models (general linear model, *P* = 0.05 or greater for all cases). Finally, it was calculated that a single straw could potentially produce 2.8 × 10^4^ oyster seed (Table 4 in the Appendix) and the average price for 6–10 mm seed (diploid) was $20 for 1000 seed or $0.02 per seed oyster (Oyster Seed Holdings Inc. 2021; University of Maryland 2022). Therefore, the calculated *gross straw value* was $560 (2.8 × 10^4^ × $0.02).

## Discussion

### Parameter effects

This study addressed the challenges of repository development in oyster aquaculture by evaluating cryopreservation needs at three different scales of production. When looking at the effect of the three parameters on both models, there was a trade-off where *throughput* improved by increasing the number of operators and the batch size, but *operating costs* also increased. *Operating costs* increased because of higher labor expenses from adding more operators and from increased supply costs from processing more oysters (higher *throughput*). To construct a productive system, these two critical factors, *throughput* and *costs*, must be balanced; *costs* should be minimized while still meeting the required *throughput* demands (Russell and Meller 2003). In this study, when the effects of the parameters were analyzed, no *throughput* requirements were set because the objective was to discover the relationships between the three parameters and the three key outputs (*throughput*, *operating costs*, and *TIS*). By setting minimum *throughput* requirements (discussed further in the “Small-scale recommendations” section), decisions can be made about how many operators or what batch size is necessary to meet *throughput* demands while minimizing *costs*.

The three parameters affected the *throughput* and *operating costs* of the baseline and enhanced models similarly, either having positive or negative relationships (Fig. 1a–f). However, in general, the magnitude of these effects was larger in the enhanced model. This could be attributed to the alternate devices implemented in the enhanced model that eliminated two major bottlenecks. These devices resulted in higher *throughput* and therefore higher *costs* in the enhanced model when compared to in the baseline model, even when all other factors remained constant between the two models (Bodenstein et al. 2022). Therefore, the enhanced model had a greater maximum potential *throughput* than the baseline model and changing certain parameters affected the enhanced model more than the baseline model.

Conversely, the *time in system* (*TIS*) of the enhanced model was not affected by the change in straws per oyster, while the *TIS* of the baseline model was affected (Fig. 1g). *Time in system* is the average amount of time it takes to process an entity moving through the system (in this study, an oyster). Industrial engineers commonly try to minimize *TIS* by eliminating wastes and bottlenecks to increase productivity (Baesler et. al. 2003; Diego Fernando and Rivera Cadavid 2007). The *TIS* of the baseline model increased rapidly (a 19-s increase for every additional straw) because one of the major bottlenecks discovered in the baseline model was sorting straws manually after freezing (Bodenstein et al. 2022). The enhanced model implemented a custom 3-D printed funnel which allowed 15 straws to be sorted at one time; therefore, the *TIS* of the enhanced model did not increase as the straws per oyster increased. The alternate devices (automated CASA software and 3-D printed funnel) implemented in the enhanced model allowed the model to handle a wider variety of situations (such as the need to process more straws per oyster) than the baseline model. Development of more flexible cryopreservation pathways, such as the enhanced model, could increase the sustainability of repositories as their needs change through time.

### Small-scale recommendations

The smallest production scale, 50 oysters per year, represented a scenario where a research laboratory was cryopreserving their own samples for future use. In this scenario, the high-throughput devices used in the enhanced model were not recommended. Using the basic equipment found in the baseline model allowed for 50 oysters to be processed in just over a week (7.3 workdays) with one operator. This time dropped to under a week (4.2 workdays) if a second operator was added. Therefore, the required number of oysters was processed within the allotted time period, without the need for high-throughput devices.

Using the high-throughput devices in the enhanced model resulted in shorter processing times than the baseline model (with 1–3 operators). This was because alternate devices (the funnel and CASA) were employed in the enhanced model to remove two major bottlenecks and increase model *throughput* (Bodenstein et al. 2022). In fact, across all scales of production, the difference between the processing times for the baseline and enhanced models remained consistent, when the number of operators was held constant. For example, with one operator, the enhanced model on average had a processing times that was 27% lower than the baseline model, across all scales of production. Both models, however, were able to process 50 oysters within the 1-year period at the smallest scale of production (Table 1). A facility that will only need to process 50 oysters a year could lower their *capital costs* by not purchasing high-throughput devices. This would slightly raise the *cost per straw* compared to a facility using high-throughput devices ($0.51 vs. $0.48 per straw, with one operator) because of the increased production time. The *breakeven price* of a straw (over a 1-year period), however, would remain lower ($85 vs. $94, with one operator, Table 1) for a facility not using high-throughput devices; therefore the high-throughput devices were not recommended. Of the two high-throughput device options, however, only the CASA caused a substantial increase in *capital cost*. The funnel was inexpensive and still increased *throughput*, even when implemented without the CASA (Bodenstein et al. 2022). Therefore, the funnel could be implemented into a production pathway operating at any scale.

While the basic equipment was sufficient for the smallest production scale, employing two operators was recommended. Within the same production scales, adjusting the number of operators affected the *percent processing failure*, required processing time, and *breakeven prices* of the baseline and enhanced models. When only one operator was assigned to a model (either model), a certain percentage of oysters were not successfully processed and “failed” out of the production system at all scales of production. However, if one more operator was assigned to a model, the failure rate dropped to zero at all scales of production. The percentage of oysters that failed to be fully processed can also be recognized as the defect rate. Products that are defective in a manufacturing system constitute one of the seven major types of waste identified in industrial engineering. Defects cause additional work, inspections, production delays, poor quality products, and increase production cost (Arunagiri and Gnanavelbabu 2014). Reducing the number of defects is imperative when creating an effective production system.

The number of operators also affected the processing times for both models and this relationship was best described by a power function. Due to the nature of power functions, as the number of operators increased from one to eight, the resulting decreases in processing times diminished. Adding one more operator (two total) had the largest effect on processing time, and adding additional operators required the same increase in labor costs without providing the same reductions in processing times. Therefore, using two operators is recommended at the small production scale to minimize *percent processing failure*, processing times, and labor costs.

### Medium-scale recommendations

The medium scale of production, 1000 oyster per year, represented a scenario where a hatchery was cryopreserving samples to back up their own lines. In this scenario, it was recommended to use the high-throughput device options in the enhanced model. Without these high-throughput devices, it took almost 150 days with one operator to process 1000 oysters. With high-throughput devices, it took just over 100 days with one operator (Table 1). This meant that a facility using the devices from the enhanced model would have faster *throughput*, a greater yearly production capacity, and higher potential profits than a facility using the basic equipment from the baseline model (Duggan 1998; Sullivan et al. 2002). Facilities, such as oyster hatcheries, interested in starting repositories should consider using higher-throughput cryopreservation pathways like the enhanced model to maximize their production capacity and profits.

At this scale of production using high-throughput equipment, for example, an operator at the hatchery would spend roughly a third of the year (100 days) cryopreserving oyster sperm. This part of the year would align with the oyster spawning season in the region (spring to early summer for *C. virginica* in the northern Gulf of Mexico, Wallace et al. 2008). This would leave the operator with the rest of the year to focus on other hatchery duties such as producing algae, conditioning broodstock, and managing the farm site. Furthermore, employing two operators at the medium-scale facility is recommended. Using an additional operator (two total) decreased the required processing time by almost half (100 to 60 days, Table 1) and decreased the *percent processing failure* to zero. This decrease in processing time and defect waste would also provide operators with more flexibility to either cryopreserve a greater number of oysters or focus on other hatchery tasks.

### Large-scale recommendations

The large scale of production, 10,000 oyster per year, represented a scenario where a commercial facility produces and cryopreserves broodstock lines to sell to laboratories and hatcheries. In this scenario, it was recommended to use the high-throughput device options and to employ six operators. Six was the minimum number of operators required to process 10,000 oysters in fewer than 260 work days based on the linear relationship between number of operators and processing time (Table 1). The average number of workdays in a year is 260 (5 work-days × 52 weeks per year) and using high-throughput device options in combination with six operators resulted in a processing time of 251, 8-h workdays (Fig. 2c). In a facility using equipment found in the baseline model, eight operators were required to attain a processing time under 260 8-h workdays. A commercial facility with faster processing times would be able to freeze their annual quota of oysters more quickly. This would leave time in the year to freeze other “in-season” aquaculture species based on an annual “spawning calendar” (Hu 2012). Using high-throughput device options would allow the facility shorten processing times and cryopreserve material from multiple species during a year providing sustained cash flow.

Processing times can be improved with a variety of methods including increasing the number of operators, switching to automated equipment, or both (Chong and Ng 2016; Edwards 1996; Kato 2000). However, labor costs can constitute the majority of *operating costs* in a system (Hu et al. 2015), making it uneconomical and inefficient to only increase the number of operators when attempting to decrease processing times. By implementing device improvements and additional operators, required processing times can decrease more quickly than when only increasing the number of operators. In addition, current operators could be trained to increase their skill and experience level. Experienced operators can perform work more quickly and efficiently (with fewer errors), therefore lowering processing times without adding more operators and increasing *operating costs* (Devotta 1983; Heneman et al. 1987; Hu et al. 2015).

Incorporating a costly piece of automated, high-throughput equipment, such as the CASA, increased the *breakeven prices* of the enhanced model as compared to the baseline model across all scales of production, despite decreasing processing times. The differences between the *breakeven prices* of the models were highest at the smallest scale of production and lowest at the highest scale of production. Therefore, the greatest benefit from incorporating an expensive, automated device was seen at the higher scales of production, highlighting the economy of scale. This principle has also been observed in previous work analyzing the economics of cryopreservation, where hatcheries investing in cryopreservation equipment saw the greatest benefits at the largest production scales (Caffey and Tiersch 2000). *Breakeven times* also followed economies of scale, with faster *breakeven times* the more oysters were produced per year (Table 1). Finally, as the proposed straw prices increased (from $15 to $60), the differences in *breakeven times* between the baseline and enhanced models decreased until they were the same at the medium and high scales of production.

This again points to the benefits of incorporating automated equipment for systems operating at high scales of production, even if initial *capital costs* are high. An important caveat, however, is that machine downtime (the proportion of time automated equipment is out of service) must be low. Frequent machine downtime creates high levels of waiting waste and decreases *throughput*, particularly at high production scales (Kumar 2014). Furthermore, to prevent machine downtime, routine machine maintenance of automated equipment must be performed (Nwanya et al. 2017). Complex equipment may require time-consuming maintenance and therefore the additional labor costs must be considered.

Finally, device improvements can be incorporated into a system without high *capital costs* by using open-source, 3-D printed hardware (Liu et al. 2021). For example, the other high-throughput device used in the enhanced model was a 3-D printed funnel that increased *throughput* without significantly increasing (< 5% difference) *capital costs* due to the low manufacturing cost of 3-D printed technology (Bodenstein et al. 2022). Another benefit of 3-D printed hardware is the ease of file sharing, making high-throughput tools accessible to a wide user base (Childress et al. 2021).

### Straw value

The *gross straw value* was calculated to be $560 per straw. In a scenario where oyster hatcheries cryopreserved and used sperm from 50 oysters (using the pathway in the baseline model), the *breakeven price* (*capital* and *operating costs* to produce one straw) would be $85 with one operator. Therefore, straws would have a net value of $475 ($560–$85) to offset *capital* and *operating costs* within 1 year. This example at the smallest production scale is a “worst-case scenario” with the highest *breakeven prices* and lowest net straw value. Due to economy of scale, *breakeven prices* decrease and *net straw value* increased at greater scales of production. For example, at the large production scale, the *net straw value* would be roughly $567 because the *breakeven prices* for both models were under $1.

Even the smallest *net straw value* ($475) was 155% higher than the highest fixed straw price used in this study ($60, Table 1). This value, however, was not as high as some of prices seen for dairy bull semen. The maximum price (or value) for a dose of semen reported in 2002 was $2019 (Carvalho et al. 2022), almost four times higher than the *net straw value* reported in this study. It should be noted that this maximum reported semen price was for a specific dairy bull (Gir), was from a proven bull (proven to produce offspring with desired characteristics relevant to milk production), and was processed to eliminate Y-chromosome-bearing sperm (increasing the probability of producing a female). These traits increased the value of the dairy bull semen and were therefore reflected in a higher price point. Even with just one of these traits, sexed semen, the price of a dose increased by more than 400% ($27 to $151, Carvalho et al. 2022).

A similar phenomena can be seen currently in the oyster industry. Straws containing sperm from oyster lines with specific traits, such as selectively bred lines or tetraploid lines, would be even more valuable and their resulting seed and could be sold for more. Research hatcheries have successfully bred lines of eastern oysters with increased resistance to major oyster pathogens, such as *Haplosporidium nelson* (MSX), Roseovarius oyster disease (ROD), and *Perkinsus marinus* (dermo) (Casas et al. 2017; Davis and Barber 1999; Haskin and Ford 1979). These pathogens caused mass mortality events in affected areas but with the advent of disease-resistant lines, oyster aquaculture has been able to expand in regions prone to disease (Proestou et al. 2016). As a result, seed oysters produced from disease-resistant lines are priced on average of 20% more than wild-type oyster seed (Ferry Cove Oyster Seed Order Form 2022; University of Maryland 2022). While these practical limitations currently exist, the use of cryopreserved sperm can add value to existing hatchery operations by allowing or easing access to genetic resources, such as disease-resistant lines, that were difficult or costly to obtain previously (Table 3).

Tetraploids are another type of high-value genetic resource that are used to produce fast-growing triploids. Triploid oysters (3N) are in high demand for many US oyster farmers due to faster growth rates and better meat quality during the summer months (Walton et al. 2012). This makes the relative value of triploid seed and tetraploid broodstock higher than diploid seed or broodstock (Table 3). The most straightforward production of triploid oysters requires use of sperm from tetraploid (4N) broodstock, which are often difficult to condition or are under proprietary control due to intellectual property constraints. In addition, creating new tetraploid lines is time-consuming and costly, making the available tetraploid genetics valuable. Cryopreservation could assist distribution of tetraploid sperm and make production of triploids easier for hatcheries. Tetraploid suppliers could distribute sperm without the downsides associated with transporting live animals and the risk of customers attempting to produce unauthorized tetraploid lines. Producing triploids results in a terminal cross and tetraploid sperm and eggs are required for the production of new tetraploids. Therefore, cryopreservation technology would facilitate greater triploid production while protecting the intellectual property of the supplier.

Transfer of cryopreserved germplasm could also ease state regulations often imposed on broodstocks entering a given state due to disease or genetic background (Tiersch and Jenkins 2001). Cryopreserved germplasm could be tested for pathogens in concert with the freezing process, meaning that producers receiving the germplasm could use it immediately and would not need to order a pathology report. Cryopreserved sperm can also open new markets for a hatchery by reducing the burden of a hatchery to maintain live adults either in coastal waters, flow-through hatcheries, or expensive recirculating aquaculture systems. Without the need to maintain all genetic lines as live animals, hatcheries would have the ability to introduce new genetics into breeding programs and produce a variety of selectively bred lines. Cryopreservation would also allow for easier distribution of genetic traits between hatcheries because cryopreserved samples can be shipped in high quantities with less risk than shipping live animals.

Finally, the service model for cryopreservation in hatcheries is yet to be determined. Hatcheries could contract a company (like the one mentioned in the large-scale scenario) to receive oysters and cryopreserve and store samples for them (referred to as a “custom collection” model in livestock). Another model would be for hatcheries to conduct cryopreservation in-house and store samples on-site or at a back-up facility, as mentioned in the medium-scale scenario. A hatchery could also offer cryopreservation services to interested parties, such as other hatcheries or researchers. Careful consideration must be given when choosing a service model as certain models would not be appropriate for all hatcheries. For example, a laboratory that only wants to cryopreserve 10 oysters once a year (similar to the small-scale scenario) would likely use a third-party company rather than investing in the equipment, personnel, and training for in-house cryopreservation. To determine the appropriate service model, future research could assess factors such as the resources, production scale, spawning schedule, biosecurity, regulatory compliance, and cryopreservation needs of hatcheries and their customers.

## Conclusions

There are unique and persistent challenges for cryopreservation and repository development of aquatic species at all scales of production. Laboratories, hatcheries, and commercial cryopreservation facilities all have different requirements to be able to freeze samples within certain time and budget constraints. Simulation modeling facilitates understanding of various cryopreservation systems and how they can be affected by key parameters, such as device options and operator capacity. Results from these models can be used to make recommendations for operating repositories in a sustainable manner, allowing them to support aquaculture enterprises in the future. Although commercial markets for cryopreserved oyster sperm are still in the early stages of development, demand will grow as frozen sperm becomes more available and cost-effective compared to traditional spawning methods (Caffey and Tiersch 2000). We indicate here that germplasm repositories can be planned and operated from the start in cost-effective ways that best serve their communities by use of simulation modeling, hastening the proliferation of cryopreservation technology.

This study also attempted to anticipate how oyster aquaculture will change in response to the integration of cryopreservation and repository storage. Hatchery production cycles may change as broodstock conditioning and “on-demand” frozen sperm make spawning more possible outside of the traditional season (Chávez-Villalba et al. 2002). Through repository storage, less labor would be needed to maintain live animals, freeing hatcheries to dedicate more resources to producing more oyster larvae, growing more algae for feeding of larvae, or developing genetically improved lines. Farmers would be able to request oysters with genetic traits suited to specific production sites as hatcheries and laboratories develop, store, and transport such genetics more easily through frozen sperm (Moore and Hasler 2017; Yang et al. 2012). With the integration of repository systems, oyster aquaculture and aquaculture in general can sustain growth as production systems reach their limits, by improving the animals that are cultured, as seen in the dairy industry.

## Figures and Tables

**Table 1:** Scaling analysis for the baseline (Model A) and enhanced (Model E) models analyzing the effect of operation scale (oysters processed per year) and the number of operators on percent processing failure, required processing time (number of 8-h workdays), cost per straw, and breakeven price. The batch size for all scenarios was five. Also reported are results for the baseline and enhanced models analyzing the effect of production scale (oysters processed per year) on breakeven time (reported in years or in days). Breakeven times were based on fixed straw prices (SP, $15, $30, and $60 per straw). The batch size for all scenarios was five and the number of operators was one

Oysters per year	Model	No. of operators	Percent processing failure (%)	Processing time (d)	Cost per straw	Breakeven price	Breakeven time (SP $15)	Breakeven time (SP $30)	Breakeven time (SP $160
50	A	1	24.3	7.3	$0.51	$85.27	5.9 y	2.9 y	1.4 y
		2	0.0	4.2	$0.51	$83.63			
		3	0.0	3.1	$0.51	$84.45			
		5	0.0	2.0	$0.54	$79.81			
		10	0.0	1.2	$0.70	$88.96			
	E	1	10.7	4.7	$0.48	$93.91	5.7 y	2.8 y	1.4 y
		2	0.0	3.2	$0.49	$94.82			
		3	0.0	2.6	$0.48	$94.82			
		5	0.0	1.9	$0.48	$98.64			
		10	0.0	1.3	$0.50	$97.67			
1000	A	1	25.6	148.2	$0.51	$4.62	103 d	51 d	26 d
		2	0.0	80.2	$0.51	$4.62			
		3	0.0	56.0	$0.51	$4.65			
		5	0.0	35.6	$0.52	$4.63			
		10	0.0	19.3	$0.56	$4.68			
	E	1	11.4	101.5	$0.49	$5.18	103 d	51 d	26 d
		2	0.0	59.5	$0.49	$5.19			
		3	0.0	43.5	$0.49	$5.19			
		5	0.0	29.3	$0.49	$5.18			
		10	0.0	17.2	$0.53	$5.21			
10,000	A	1	25.0	1479.5	$0.51	$0.92	11 d	4 d	4 d
		2	0.0	800.6	$0.51	$0.92			
		3	0.0	559.0	$0.51	$0.92			
		5	0.0	355.5	$0.52	$0.93			
		10	0.0	192.4	$0.55	$0.96			
	E	1	11.1	1021.1	$0.49	$0.95	11 d	4 d	4 d
		2	0.0	593.2	$0.49	$0.96			
		3	0.0	431.7	$0.49	$0.96			
		5	0.0	289.3	$0.49	$0.96			
		10	0.0	168.1	$0.53	$1.00			

**Table 2:** Linear regression results for the baseline and enhanced models analyzing the effect of three parameters (straws per oyster, batch size, and number of operators) on four output statistics (throughput, time in system (TIS), operating cost, and cost per oyster). Bolded *P* values are significant

Model	Output statistic	Parameter	Estimate	*t* value	*P* value	*R*^*2*^ value	Figure reference
A	Throughput	Straws per oyster	− 0.03	− 11.36	< **0.01**	0.88	Figure 1a
		Batch size	1.30	4.12	**0.01**	0.77	Figure 1b
		No. operators	15.32	13.17	< **0.01**	0.98	Figure 1c
	Operating cost ($)	Straws per oyster	2.14	26.32	< **0.01**	0.98	Figure 1d
		Batch size	11.37	4.71	**0.01**	0.82	Figure 1e
		No. operators	515.82	25.47	< **0.01**	0.99	Figure 1f
	TIS (h)	Straws per oyster	0.01	9.67	< **0.01**	0.85	Figure 1g
		Batch size	0.41	4.52	< **0.01**	0.80	Figure 1h
		No. operators	− 0.05	− 1.10	0.33	0.23	Figure 1i
	Cost per oyster ($)	Straws per oyster	0.24	1596.30	< **0.01**	1	–-
		Batch size	− 2.47	− 3.88	**0.01**	0.75	–-
		No. operators	0.11	3.60	**0.02**	0.76	–-
E	Throughput	Straws per oyster	− 0.018	− 10.38	< **0.01**	0.86	Figure 1a
		Batch size	2.12	4.13	**0.01**	0.77	Figure 1b
		No. operators	20.39	14.75	< **0.01**	0.98	Figure 1c
	Operating cost ($)	Straws per oyster	4.73	54.90	< **0.01**	0.99	Figure 1d
		Batch size	27.18	6.20	< **0.01**	0.88	Figure 1e
		No. operators	626.34	24.68	< **0.01**	0.99	Figure 1f
	TIS (h)	Straws per oyster	0.00	0.64	0.53	0.02	Figure 1g
		Batch size	0.33	5.29	< **0.01**	0.85	Figure 1h
		No. operators	− 0.12	− 1.67	0.17	0.41	Figure 1i
	Cost per oyster ($)	Straws per oyster	0.23	2317.20	< **0.01**	1	–-
		Batch size	− 2.48	− 3.71	**0.01**	0.73	–-
		No. operators	0.07	2.41	0.07	0.59	–-

**Table 3:** Hypothetical relative values for broodstock, juvenile oysters sold at hatcheries (oyster “seed”), and oyster sperm (frozen in straws) possessing a variety of genetic traits. Provenance indicates if the location of origin or pedigree of the broodstock is known. Relative values of broodstock and frozen sperm were assessed based on the perspective of oyster hatchery managers (who would sell the resulting seed to producers), and relative values of seed were assessed based on the perspective of commercial oyster producers (i.e., farmers). “NA” (not available) indicates that triploid broodstock do not exist and that sperm of triploids (3 sets of chromosomes; 3N) is not cryopreserved because these animals are functionally sterile and are typically produced by crossing tetraploid (4N) and diploid (2N) broodstocks. “NA” also indicates that tetraploid seed are typically not made available to oyster producers. The “$” symbols only indicate relative value levels, i.e., a spaces with two “$” symbols have greater value but not twice the value of spaces with one “$” symbol

Source and genetic traits	Known provenance	Relative value as broodstock	Relative value as frozen sperm	Relative value as seed
2N	No	$	$	$
3N	No	NA	NA	$$
4N	No	$$	$$	NA
2N	Yes	$$	$$	$$
3N	Yes	NA	NA	$$$
4N	Yes	$$$	$$$	NA
Disease-resistant 2N	Yes	$$$	$$$	$$$
Disease-resistant 3N	Yes	NA	NA	$$$$
Disease-resistant 4N	Yes	$$$$	$$$$	NA
Low salinity 2N	Yes	$$$	$$$	$$$
Low salinity 3N	Yes	NA	NA	$$$$
Low salinity 4N	Yes	$$$$	$$$$	NA

## Data Availability

The Simio models used in this study will be available on the website of the Aquatic Germplasm and Genetic Resources Center (aggrc.com).

## Appendix

**Table 4:** For each major life stage of an oyster, the time after fertilization, percent fertilization, percent survival rate, estimated number of that life stage, and estimated number that could be produced from one straw of cryopreserved semen are reported. The number of eggs reported (2 × 10^8^) were from 10 females each producing 2 × 10^7^ eggs. The fertilization and survival rates were based on published work (Wallace et al. 2008), and on discussions with hatchery operators (B. Callam pers comm at the LSU hatchery; F.S. Rikard pers comm at the Auburn hatchery; W.C. Walton pers comm at the VIMS hatchery)

Life stage	Time after fertilization	Percent fertilization	Percent survival	Estimated number at each life stage	Number produced from one straw
Eggs	NA	20%	–-	2 × 10^8^	–-
Larvae	1 d	–-	16%	4 × 10^7^	6.7 × 10^5^
Pediveligers	14 d	–-	33%	6.4 × 10^6^	1.1 × 10^5^
Spat	14–17 d	–-	80%	2.1 × 10^6^	3.5 × 10^4^
Seed (R8)	3–4 months	–-	70%	1.7 × 10^6^	2.8 × 10^4^
Adults	1 yr	–-	–-	1.2 × 10^6^	2.0 × 10^4^
